# Templating effect of neutral dithiane ligands on the self-assembly of cationic Au(I)-phosphine units

**DOI:** 10.55730/1300-0527.3789

**Published:** 2026-03-30

**Authors:** Farzane OSANLOU, Abdollah NESHAT, Mohammad Reza YOUSEFSHAHI, Vaclav EIGNER, Michal DUSEK, Shahram SEIDI

**Affiliations:** 1 Department of Chemistry, Khaje Nassir Toosi University of Technology, Tehran, Iran; 2 Department of Chemistry, Institute for Advanced Studies in Basic Sciences, Zanjan, Iran; 3 Institute of Physics of the Czech Academy of Sciences, Prague, Czech Republic

**Keywords:** Au(I) complexes, diphosphine ligands, dithiane, templating effect, aurophilic interactions, single-crystal X-ray diffraction

## Abstract

The interactions between the dinuclear Au(I) precursor (dppm)Au_2_Cl_2_ and neutral sulfur-containing dithianes (specifically 1,3- and 1,4-dithiane) were examined in the presence of NH_4_PF_6_. Although these sulfur-based ligands were initially employed to facilitate the formation of mixed phosphine/sulfur Au(I) complexes, single-crystal X-ray diffraction analysis revealed the unexpected generation of multinuclear Au(I) assemblies exclusively coordinated by phosphine ligands. When 1,3-dithiane was present, a trinuclear Au(I) complex was isolated, characterized by a central four-coordinate Au(I) center exhibiting a distorted see-saw geometry, flanked by two terminal Au(I) centers with T-shaped coordination environments. Importantly, the dithiane ligand did not coordinate directly to the gold centers in the final complex; rather, it functioned as a neutral structural template that directed the self-assembly of the trinuclear aggregate. Conversely, in the absence of dithiane, a structurally distinct trinuclear Au(I) isomer was obtained, underscoring the significant influence of donor additives in modulating aggregation pathways and determining the ultimate molecular geometries. The observed short Au(I)···Au(I) distances suggest the presence of substantial intramolecular aurophilic interactions, which play a crucial role in stabilizing these assemblies. Collectively, these findings illustrate that minor variations in reaction parameters, as well as the inclusion or exclusion of weak, noncoordinating donor ligands, can markedly affect the self-assembly processes and structural diversity of multinuclear Au(I) complexes.

## 1. Introduction

A notable consequence of relativistic effects is the contraction and stabilization observed in the s and p orbitals, contrasted by the expansion and destabilization of the d and f orbitals. The enlarged and more diffuse 5d orbitals engage in mutual interactions, giving rise to noncovalent forces within dicationic gold dimers. This phenomenon, termed “aurophilicity,” in Au(I) complexes is contingent upon the spatial proximity of the Au(I) centers and is significantly modulated by the characteristics and spatial arrangement of the coordinating ligands [1–3]. For this reason, Au(I) complexes remain a focal point of research. These characteristics allow Au(I) centers to assume coordination geometries that diverge from the idealized linear configuration generally observed in d^10^ metal ions, thereby facilitating the formation of structurally diverse multinuclear assemblies. Such complexes have been employed in various domains, including homogeneous catalysis, photophysical materials, and medicinal chemistry, where minor modifications in the ligand environment can lead to substantial variations in both structure and function [4–8].

Diphosphine ligands, exemplified by bis(diphenylphosphino)methane (dppm), are notably effective in stabilizing polynuclear Au(I) complexes owing to their capacity to bridge metal centers and promote short Au(I)···Au(I) distances. Such interactions frequently play a crucial role in guiding the aggregation of Au(I) units and in stabilizing atypical coordination geometries, including T-shaped, trigonal-planar, and tetrahedral configurations [9–12]. Consequently, Au(I) complexes bridged by dppm have been extensively studied as model systems for investigating aurophilic interactions and their structural implications.

Conversely, sulfur-containing ligands, including thioethers and dithianes, are typically regarded as weak donors to Au(I) centers. Although these ligands can coordinate to soft metal ions under suitable conditions, their function in Au(I) chemistry may transcend direct metal–ligand coordination. Notably, flexible dithiane ligands, capable of adopting various conformations, can modulate metal assembly processes via secondary interactions or spatial confinement effects, even in the absence of robust coordination [13].

Inspired by the well-documented effectiveness of mixed phosphine/sulfur ligand frameworks in transition metal chemistry and catalysis [14–18], our initial objective was to synthesize heteroleptic Au(I) complexes incorporating both phosphine and sulfur donor ligands, employing straightforward Au(I) precursors. However, during these studies, we found that reactions of (dppm)Au_2_Cl_2_ with neutral dithiane ligands in the presence of a halide abstractor did not produce the anticipated mixed phosphine–sulfur complexes. Rather, the reactions unexpectedly yielded multinuclear Au(I) complexes coordinated exclusively by phosphine ligands.

Single-crystal X-ray diffraction and solution NMR analyses revealed that the dithiane ligands did not engage in direct coordination with the gold centers in the resulting compounds. Rather, these ligands functioned as neutral structure-directing templates that modulated the self-assembly and rearrangement processes of cationic Au(I) phosphine complexes. Notably, the inclusion or exclusion of dithiane significantly influenced the nuclearity, geometric configuration, and solution-phase stability of structurally related trinuclear Au(I) aggregates, which were further stabilized by intramolecular aurophilic interactions.

This study offers a comprehensive structural and spectroscopic analysis of multinuclear Au(I) assemblies, with a particular focus on the templating role of weak, noncoordinating sulfur donors and their impact on the self-assembly processes of Au(I) species. By establishing correlations between solid-state structural data and solution-phase behavior, the investigation clarifies how minor variations in reaction parameters and the presence of weak donor additives can direct the selective formation of different isomeric Au(I) compounds.

## 2. Materials and methods

Reagents and solvents were used as received from commercial suppliers. NMR spectra in solution were recorded on a DPX 400 MHz spectrometer (Bruker, Billerica, MA, USA) in acetone-*d*_6_ or CDCl_3_ with SiMe_4_ (for ^1^H and ^13^C) and H_3_PO_4_ (for ^31^P) as external references. (dppm)Au_2_Cl_2_, (dppe)Au_2_Cl_2_, and (IPr)AuCl were synthesized according to the literature procedures [19–22].

### 2.1. Synthesis of 1 and 2

(dppm)Au_2_Cl_2_ (50 mg, 0.06 mmol) was dissolved in a mixture of dichloromethane and methanol, and to that solution was added ammonium hexafluorophosphate (19 mg, 0.12 mmol) dissolved in 1 mL of methanol, followed by the addition of 1,3-dithiane (7 mg, 0.029 mmol). The reaction was continued for 2 h. The solvent was then removed and the precipitates were dissolved in dichloromethane. After the removal of the ammonium chloride byproduct, the obtained clear solution (2 mL) was crystallized by adding acetonitrile (1 mL) and a few drops of diethyl ether. Yield: 25 mg, 54%. ^1^H NMR (CD_2_Cl_2_, 400 MHz): δ 7.71–7.65 (m, 8H), 7.55–7.50 (m, 6H), 7.46–7.40 (m, 8H), 3.83 (t, ^2^*J_HP_* = 12 Hz, 2H). ^13^C NMR (CD_2_Cl_2_, 101 MHz) δ 133.5, 133.4, 132.5, 129.5, 129.4, 129.3, 128.3, 128.0, 127.7, 26.8. ^31^P NMR (CD_2_Cl_2_, 162 MHz) δ 35.8 (d, ^2^*J*_PP_ = 32 Hz), 35.6 (d, ^2^*J*_PP_ = 32 Hz), 29.5 (d, ^2^*J*_PP_ = 32 Hz), 29.2 (d, ^2^*J*_PP_ = 32 Hz). Complex **2** was synthesized using a similar procedure but 1,3-dithiane was not used.

### 2.2. Synthesis of 5

The dppm (15 mg, 0.04 mmol) was completely dissolved in methanol, and to that solution was added ammonium hexafluorophosphate (13 mg, 0.08 mmol) followed by the addition of (IPr)Au-Cl (50 mg, 0.08 mmol). The reaction mixture was stirred for 2 h at room temperature. No precipitate formation was observed during the reaction. The solvent was then removed under reduced pressure and the remaining precipitates were dissolved in dichloromethane solvent and centrifuged until the ammonium chloride byproduct was separated. The obtained clear solution was crystallized by adding methanol and a few drops of diethyl ether. Yield: 30 mg, 55%. Only peaks related to **5** are provided. ^1^H NMR (DMSO-*d*_6_, 400 MHz): δ 8.19 (s, 1H), 8.05 (s, 2H), 7.93–7.90 (m, 2H), 7.66–7.55 (m, 4H), 7.47–7.40 (m, 7H), 7.23–7.21 (m, 1H), 7.03 (t, 2H), 6.81–6.76 (m, 2H), 2.15 (m, 2H). ^13^C NMR (DMSO-*d*_6_, 101 MHz) δ 187.2, 185.9, 173.3, 145.9, 145.8, 145.6, 145.1, 135.0, 134.5, 134.0, 133.7, 133.2, 133.0, 132.7, 131.5, 131.4, 131.0, 131.0, 129.8, 129.6, 128.9, 127.0, 126.6, 126.3, 126.2, 125.0, 124.8, 124.5, 28.8, 24.5, 24.4, 24.1, 24.0. ^31^P NMR (DMSO-*d*_6_, 162 MHz) δ 35.8, 28.9, −l144.2.

### 2.3. X-ray crystal structure determination and refinement

Crystals of **1** were grown from a diffusion of diethyl ether into a mixture of dichloromethane and acetonitrile solutions. The X-ray diffraction data of **1**, **5**, and dppf-dithiane were collected at 95 K with an OD Supernova diffractometer (Rigaku, Tokyo, Japan) using the Atlas S2 CCD detector and mirror-collimated radiation from a sealed microfocus X-ray tube (λ = 0.71073 Å for **1** and λ = 1.54184 Å for **5** and dppf-dithiane). The X-ray diffraction data of **1** were collected at 120 K with an OD Gemini diffractometer (Rigaku) using the Atlas S2 CCD detector and a classical sealed X-ray tube with graphite monochromator (λ = 0.71073 Å). Integration of the CCD images, absorption correction, and scaling were done using CrysAlisPro 1.171.41.123a (Rigaku). Crystal structures were solved by charge flipping with the program SUPERFLIP [23] and the structures of **6** and dppf-dithiane were refined with Jana2020 [24], while the structure of **5** was refined using Crystals [25]. The hydrogen atoms were discernible in residual electron density maps and could be refined to a reasonable geometry, but according to common practice, they were maintained at ideal positions with *U*_iso_ kept at 1.2 *U*_eq_(C). In the crystal structure of **5**, the gold cation was weakly disordered. The gold cation was refined with soft restraints on anisotropic displacement parameters (ADPs) and hard restraints on the sum of occupancies, resulting in a final occupancy ratio of 962(11):37(11). The methanol molecule was also found to be disordered, and it was refined with soft restraints on geometry and ADPs and hard restraints on the sum of occupancies, resulting in a final occupancy ratio of 60(2):40(2). The hydrogen of the methanol hydroxy group was not visible in the difference electron density maps; therefore, it is absent in the structure model. The crystal structure of dppf-dithiane was found to be twinned, with the twin volumes refined to 0.8216(18) and 0.1784(18). The molecular structure plots were prepared with Diamond 3.0 [26]. Crystallographic data, details of the data collection, structure solution, and refinements are listed in Supplementary Tables S1 and S2.

## 3. Results and discussion

**Scheme 1:**
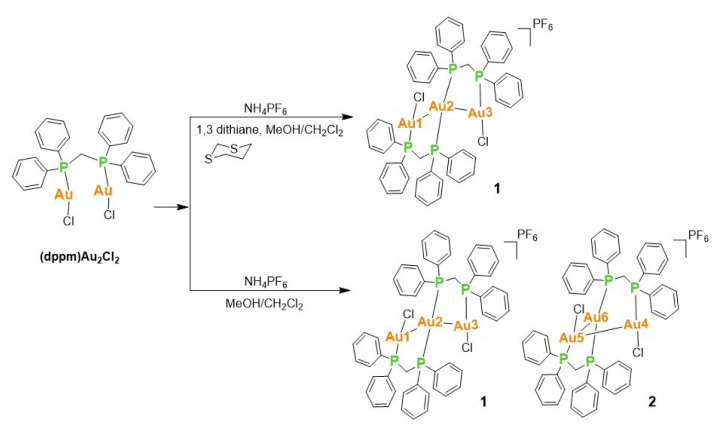
Reaction conditions for the synthesis of trinuclear Au(I) complexes **1** and **2** starting with (dppm)Au_2_Cl_2_ in the presence and absence of 1,3-dithiane.

Dithiacyclohexanes with boat and chair conformations are flexible neutral thioethers that have been reported to exhibit diverse structural roles in coordination chemistry, ranging from weak or transient coordination to noncovalent structure-directing effects [13]. To explore the influence of neutral sulfur-containing additives on the aggregation behavior of cationic Au(I)-phosphine species, the nucleophilic substitution of chloro ligands in (dppf)Au_2_Cl_2_, (dppm)Au_2_Cl_2_, and (dppe)Au_2_Cl_2_ was carried out using selected dithiacyclohexanes such as 1,3- and 1,4-dithiane in the presence of NH_4_PF_6_. The reaction of (dppm)Au_2_Cl_2_ with 1,3-dithiane gave an unprecedented trinuclear Au(I) structure, **1**, in which the central Au(I) atom adopts a four-coordinate, distorted see-saw geometry, while the two terminal Au(I) atoms are three-coordinate and best described as T-shaped (Scheme 1).

**Figure 1:**
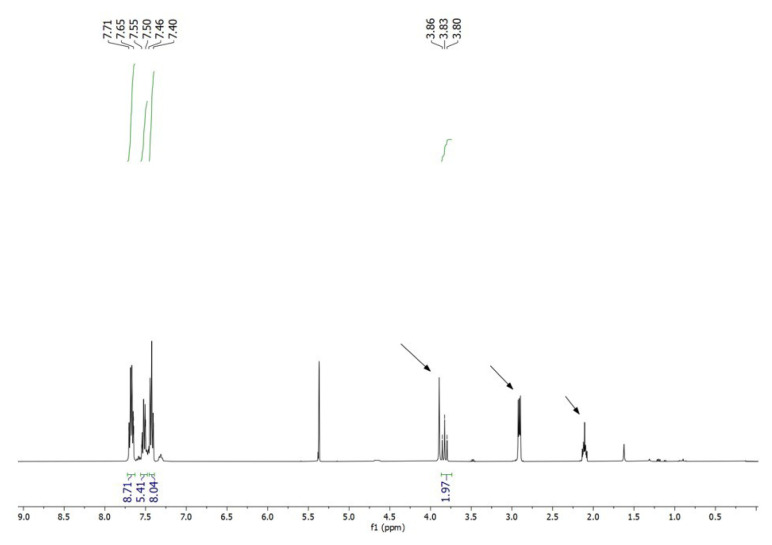
^1^H NMR spectrum of **1** in CD_2_Cl_2_. Signals indicated by arrows correspond to free 1,3-dithiane. Singlet signals at ~1.6 ppm and ~5.4 ppm are attributed to traces of water and dichloromethane solvent.

**Figure 2:**
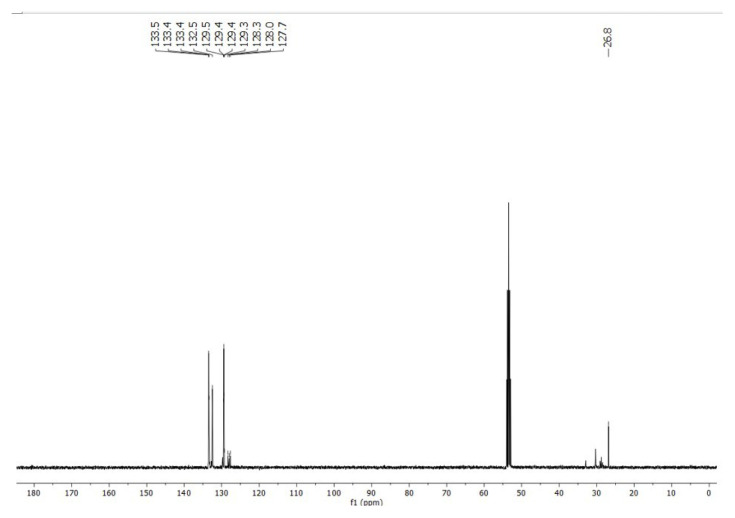
^13^C NMR spectrum of **1** in CD_2_Cl_2_. Multiplet signal at ~53 ppm belongs to CD_2_Cl_2_.

**Figure 3:**
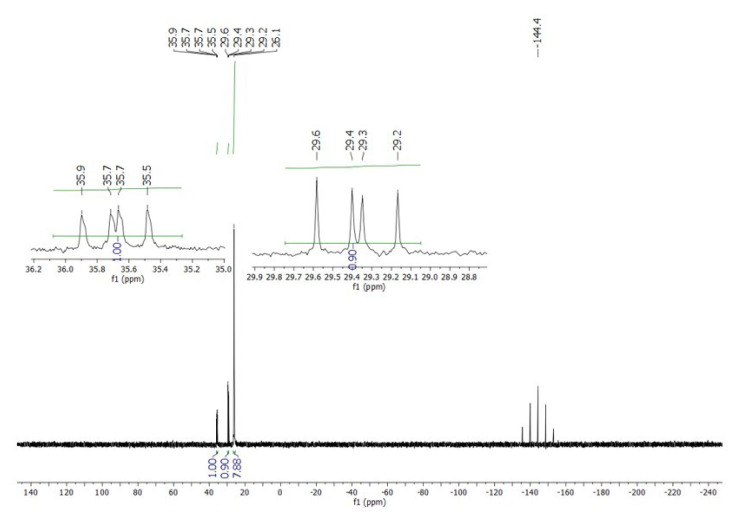
^31^P NMR spectrum of **1** in CD_2_Cl_2_. The sharp signal at 26.1 ppm belongs to the (dppm)Au_2_Cl_2_ precursor.

**Figure 4:**
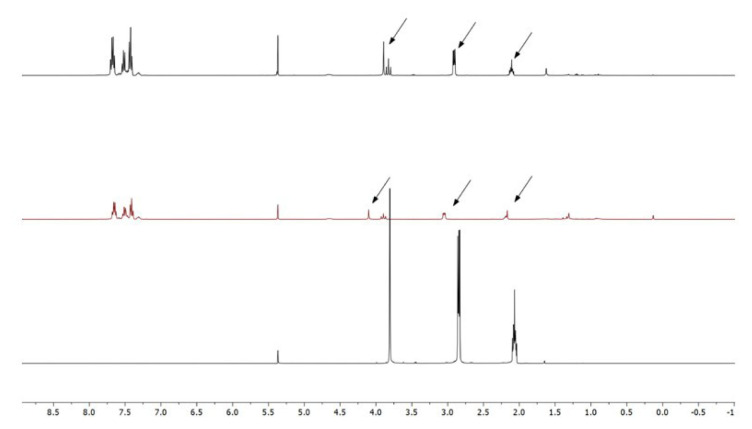
From bottom to top: ^1^H NMR spectrum of free 1,3-dithiane in CD_2_Cl_2_, ^1^H NMR spectrum of coordinated 1,3-dithiane upon reaction for 20 min with (dppm)Au_2_Cl_2_, and ^1^H NMR spectrum of noncoordinated 1,3-dithiane after 2 h of reaction.

**Figure 5:**
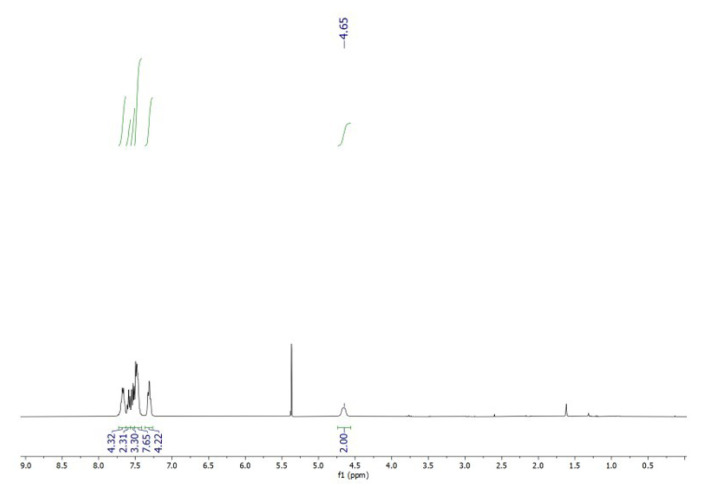
^1^H NMR spectrum of **2** in CD_2_Cl_2_. Peaks at ~1.6 ppm and ~5.4 ppm belong to traces of water and dichloromethane.

**Figure 6:**
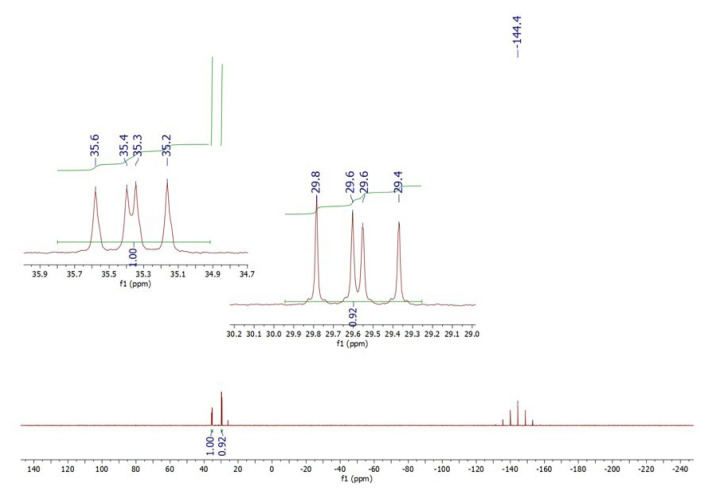
^31^P NMR spectrum of **2** in CD_2_Cl_2_.

In a concentrated solution, colorless crystals of **1** precipitate out first, allowing its structural characterization. Whereas the single-crystal X-ray diffraction data of **1** provide undisputable structural information, based on the solution ^1^H NMR and ^13^C NMR (Figures 1 and 2), the solid-state structure of **1** cannot be preserved in solution and it is in equilibrium with the (dppm)Au_2_Cl_2_ precursor, as evidenced by the presence of a sharp ^31^P signal at 26.1 ppm (Figure 3). The ^1^H NMR spectrum of the reaction depicted in Scheme 1 was recorded in 20 min and after 2 h (Figure 4). A comparison with the free 1,3-dithiane signals suggests a transient interaction of this molecule with the cationic [(dppm)Au_2_]^2+^ species (middle spectrum in Figure 4) generated upon the addition of the NH_4_PF_6_. However, unlike the previous experiment with (dppf)Au_2_Cl_2_, this transient interaction ultimately promotes the formation of a trinuclear Au(I) assembly rather than a sulfur-coordinated product. In the ^1^H NMR spectrum of **1**, the triplet resonance observed at 3.83 ppm (^2^*J*_HP_ = 12 Hz) belongs to the methylene protons of the dppm ligand, which correlates well with the literature data for similar structures in which a metallacycle involving a dppm ligand has been formed [11,12,27]. The ^31^P NMR data of **1** displayed the expected doublet of doublets pairs for the phosphorus atoms. The downfield shifted doublet signals centered at 35.8 and 35.6 ppm are assignable to the trans phosphorus atoms coordinated to the central Au(I) atom. The upfield-shifted second doublet pair at 29.5 and 29.3 ppm belong to the phosphorus atoms situated trans to the chlorine atoms. To demonstrate the importance of the 1,3-dithiane molecule in the generation of **1**, a similar reaction to that depicted in Scheme 1 was carried out in the absence of 1,3-dithiane. The ^1^H NMR of the product of this reaction in Figure 5 shows a methylene signal at 4.65 ppm, which is significantly downfield-shifted compared to the methylene signal observed for **1** at 3.83 ppm. The ^31^P NMR spectrum of this new compound (**2** in Scheme 1) in Figure 6 shows, in addition to the similar multiplet signal patterns observed in the case of **1**, a new set of multiplets in the 35.2 to 35.6 ppm range. Furthermore, the free (dppm)Au_2_Cl_2_ signal is absent this time, indicating the stability of the new complex in solution.

**Figure 7:**
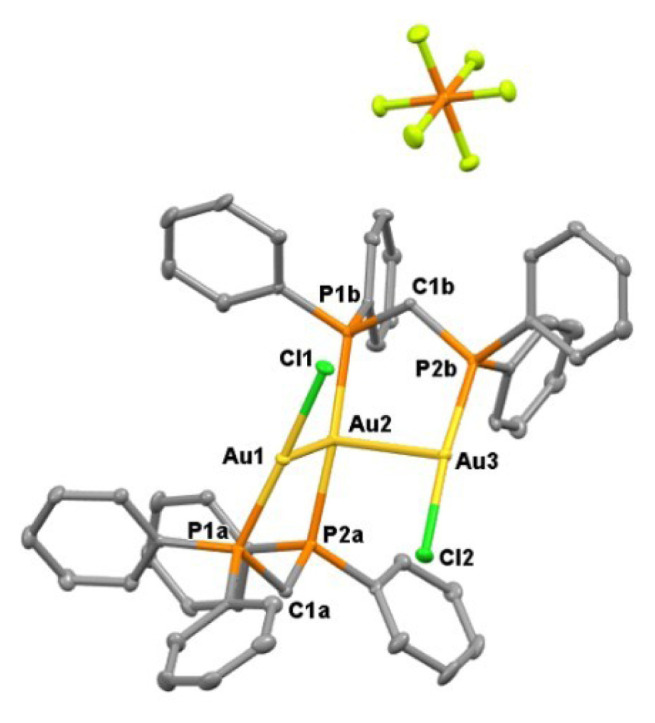
X-ray crystal structure of **1** showing thermal ellipsoids for nonhydrogen atoms at 30% probability level. Hydrogen atoms are omitted for clarity. Selected bond lengths [Å] and bond angles [°]: Au1–P1a 2.2351(12), Au2–P2a 2.3125(12), Au2–P1b 2.3102(12), Au3–P2b 2.2370(13), Cl1–Au1–P1a 174.94(4), P2a–Au2–P1b 165.68(4), Cl2–Au3–P2b 174.11(4), Au1–P1a–C1a 112.20(15), Au1–P1a–C2a 111.80(15), Au1–P1a–C8a 114.69(16) Au2–P2a–C1a 115.24(14), Au2–P2a–C14a 109.58(16).

**Figure 8:**
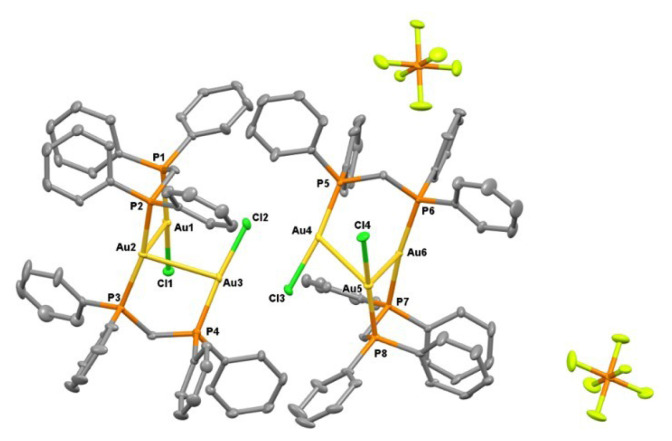
X-ray crystal structures of **1** (molecule on the right) and **2** (molecule on the left) with thermal ellipsoids for nonhydrogen atoms at 30% probability level. Hydrogen atoms are omitted for clarity. Selected bond lengths [Å] and bond angles [°] for the molecule on right-hand side: Au4–Au5 3.1562(15), Au4–Au6 3.2573(18), Au4–Cl3 2.3091(17), Au4–P5 2.2442(16), Au5–Au6 3.2063(3), Au5–Cl4 2.3015(14), Au5–P8 2.2415(13), Au6–P6 2.3057(13), Au5–Au4–Au6 59.965(12), Cl4–Au5–P8 170.72(4), P6–Au6–P7 169.41(4), Cl3–Au4′–P5 163.2(5), Au6–P7 2.3022(13).

Single crystals of **1** were obtained in three solvent mixtures, including dichloromethane, acetonitrile, and diethyl ether. As shown in Figure 7, this revealed a trinuclear complex featuring a central Au2 and terminal T-shaped gold(I) centers (Au1, Au3). For a four-coordinate Au2 center, the geometry parameter τ_4_ in complex **1** is 0.665, confirming a distorted seesaw geometry [28]. The intramolecular distance between the Au1 and Au3 gold(I) centers on each side is 3.5330(3) Å, indicating Au···Au contacts that are longer than those typically associated with strong aurophilic interactions. However, the distances of Au2–Au1 and Au2–Au3 are 3.0593(3) and 3.1082(3) Å, respectively. Considering the sum of two van der Waals radii of a gold atom (3.80 Å), the observed contacts indicate a significant intramolecular aurophilic interaction [29,30]. Owing to their important physical and chemical significance, aurophilic interactions have been the subject of both experimental investigations, such as wave function-based crystallographic methods, and theoretical studies [31]. The covalent bond lengths of Au1–P1a and Au3–P2b are 2.2351(12) and 2.2370(13) Å, which are shorter than the Au2–P2a and Au2–P1b distances of 2.3125(12) and 2.3102(12) Å, respectively, indicating trans phosphorus atoms weakly coordinating to the central Au(I) atom. The Cl1–Au1–P1a and Cl2–Au3–P2b bond angles of 174.94(4)° and 174.11(4)° also show a more linear arrangement of atoms than that in P2a–Au2–P1b with a less obtuse angle of 165.68(4)°. As noted previously, the ^1^H NMR spectrum of the product of the bottom reaction depicted in Scheme 1, in the absence of 1,3-dithiane, revealed a different pattern than that discussed for **1**. Fortunately, suitable single crystals of **2** were obtained in dichloromethane and diethyl ether. As shown in Figure 8, the asymmetric unit of **2** contains two structural isomers. The first is similar to **1**, where the central gold atom (Au2 in Figure 2) adopts a distorted see-saw geometry (τ_4_ = 0.646). The second molecule, **2**, is a trinuclear complex featuring a central tetracoordinate Au(I) center and terminal T-shaped Au(I) centers. The geometry parameter, τ_4_, for Au6 in complex **2** is 0.654. Therefore, the coordination of the central Au(I) in all three structures exhibits a distorted see-saw geometry. In compound **2**, the Au6 atom, which is analogous to Au2 in compound **1** and has two phosphorus ligands in the trans positions, is located at distances of 3.257 Å and 3.206 Å from Au4 and Au5, respectively. Au4 and Au5, on the other hand, have a bond length of 3.154 Å. Compared to compound **1**, where the distance between Au1 and Au3 is 3.393 Å, the analogous Au4 and Au6 centers in compound **2** exhibit stronger aurophilic interactions. While understanding the nature of bonding in these molecules requires further theoretical investigation, the characteristics of the closed-shell Au(I)···Au(I) interaction have been debated within the scientific community. Hoffmann and colleagues, utilizing extended Hückel calculations, proposed that this atypical interaction results from the hybridization of gold’s 5d orbitals with its 6s and 6p orbitals [32]. Conversely, employing the Hartree–Fock method, no indication of closed-shell attraction in the perpendicular (ClAuPH_3_)_2_ complex has been found [33]. However, the presence of attraction was detected using the second-order Møller–Plesset perturbation theory, implying that Au(I)···Au(I) aurophilic interactions primarily arise from electron correlation effects [34]. Subsequent investigations have identified dispersion forces as the dominant component of this interaction. Recent studies on trans (AuX)_2_ complexes, where X represents a halogen ligand in gold(I)-halogen systems, reveal a short Au–Au distance characteristic of covalent bonding, yet the interaction energy remains weak, consistent with noncovalent interactions. It is now widely accepted that polarization and electronic correlation effects constitute the principal contributors to this interaction at the post-Hartree–Fock theoretical level [35]. Furthermore, research on the Au_2_F_2_ complex, which adopts a zigzag conformation, indicates that the d^10^–d^10^ closed-shell interaction between AuF monomers exhibits behavior analogous to a coordinate covalent bond [36]. The ^1^H and ^31^P NMR spectra shown in Figures 5 and 6 are consistent with, and can reasonably be attributed to, this isomeric form. In addition, a careful analysis of ^1^H NMR and single-crystal analysis of the top reaction in Scheme 1 reveals that the presence of 1,3-dithiane directs the formation of **1** in solution, which exists in equilibrium with the starting Au_2_(dppm)_2_Cl_2_ precursor. A weak signal at 4.65 ppm in the ^1^H NMR spectrum of **1** in Figure 1 also suggests the presence of a small amount of **2**. On the other hand, in the absence of 1,3-dithiane, **2** is solely formed in the solution phase. The crystallization of **1** alongside **2** in concentrated dichloromethane reveals that both isomers are energetically very close.

**Figure 9:**
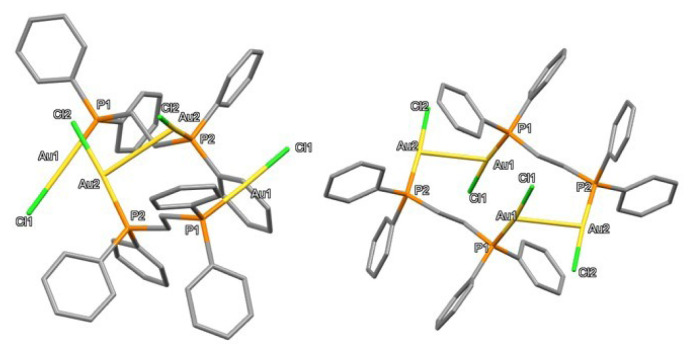
X-ray crystal structures of **3** (right) and **4** (left).

**Scheme 2:**
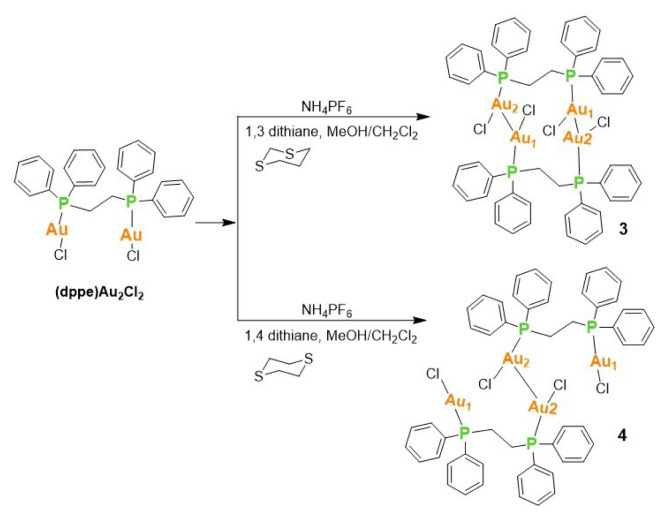
Reaction conditions for the synthesis of trinuclear Au(I) complexes **3** and **4** starting with (dppe)Au_2_Cl_2_ in the presence of 1,3-dithiane and 1,4-dithiane.

Attempts to derivatize (dppe)Au_2_Cl_2_ using sulfur donor ligands, as shown in Scheme 2, did not yield the intended product. Instead, a cyclic tetramer consisting of four Au(I) atoms and dppe ligands was formed in the reaction with 1,3-dithiane (the right molecule in Figure 9). A linear chain of Au(I) atoms and dppe ligands was obtained when a similar reaction was carried out using 1,4-dithiane. The crystal structures of these compounds have already been published (CCDC numbers: 1478422 and 1143887) [37,38]. The crystal structure of **3** shows that it is a classic dinuclear gold(I) complex bridged by a diphosphine ligand. The central unit is an eight-membered ring formed by two gold atoms (Au1, Au2) and the two phosphorus atoms (P1, P2) of the bridging ligand 1,2-ethanediylbis(diphenylphosphine) (often called dppe). The connecting ethyl group is defined by atoms C13 and C14. Each gold center is further coordinated by a chloride ligand (Cl1 for Au1, Cl2 for Au2), completing a linear, two-coordinate geometry that is typical for gold(I). The P-Au-Cl angles are expected to be very close to 180°, consistent with linear coordination at Au(I). The space group *P*2_1_/*n* is centrosymmetric, meaning that the asymmetric unit contains one half of the dinuclear complex, and the full molecule is generated by the inversion center. The core structure of **4** is essentially identical to **3**, a dinuclear gold(I) complex with a dppe bridging ligand. Each gold center (Au1, Au2) is in a linear, two-coordinate geometry, bonded to one phosphorus atom from the dppe ligand and one chloride ligand (Cl1, Cl2).

**Figure 10:**
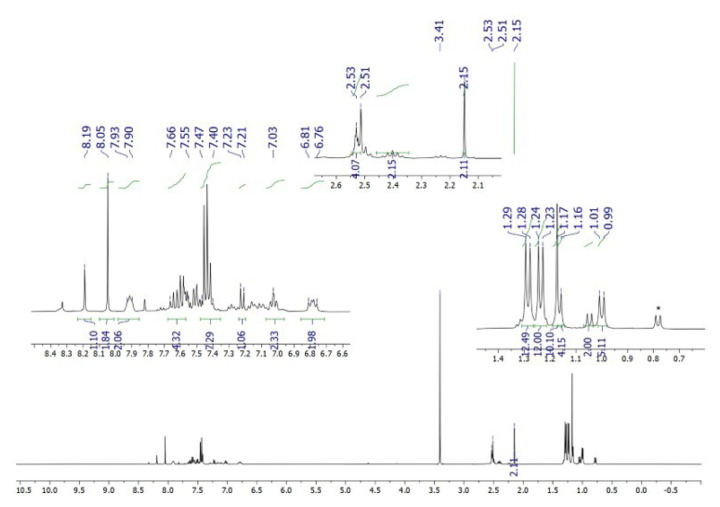
^1^H NMR spectrum of **5** (synthesized in methanol) in DMSO-*d*_6_. Asterisk denotes [IPr_2_Au]^+^ species. Signals at ~2.5 ppm and ~3.5 ppm belong to traces of water and protio-solvent.

**Figure 11:**
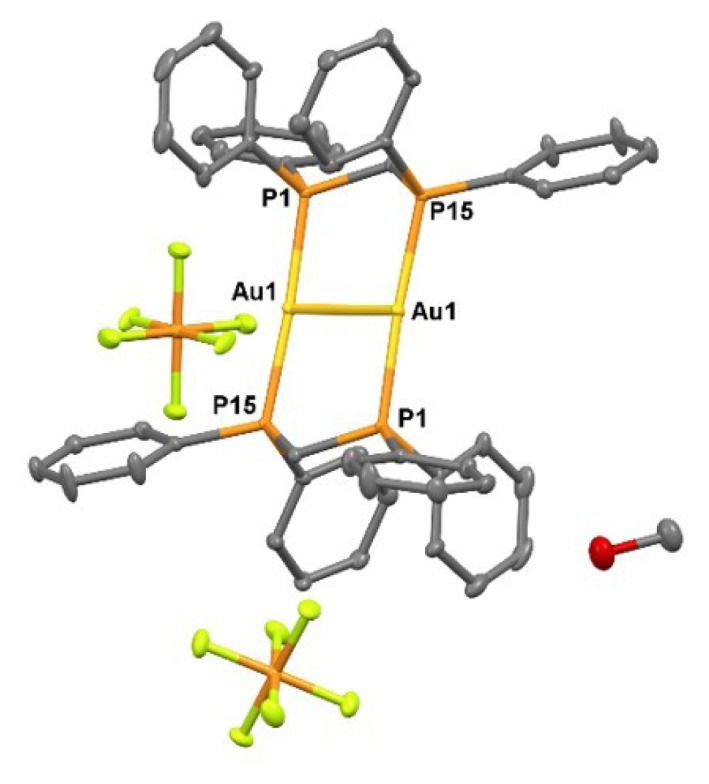
X-ray crystal structure of **5** showing thermal ellipsoids for nonhydrogen atoms at 30% probability level. Hydrogen atoms are omitted for clarity. Selected bond lengths [Å] and bond angles [°]: Au1–Au1 2.951(3), Au1–P15 2.3130(13), Au1–P1 2.3129(13), Au2–Au2 2.75(3), Au2–P15 2.25(2), Au2–P1 2.43(3), Au1–Au1–P15 93.36(6), Au1–Au1–P1 89.09(5), P15–Au1–P1 176.07(7), Au2–Au2–P15 99.2(12), Au2–Au2–P1 86.8(14), P15–Au2–P1 162(2).

**Figure 12:**
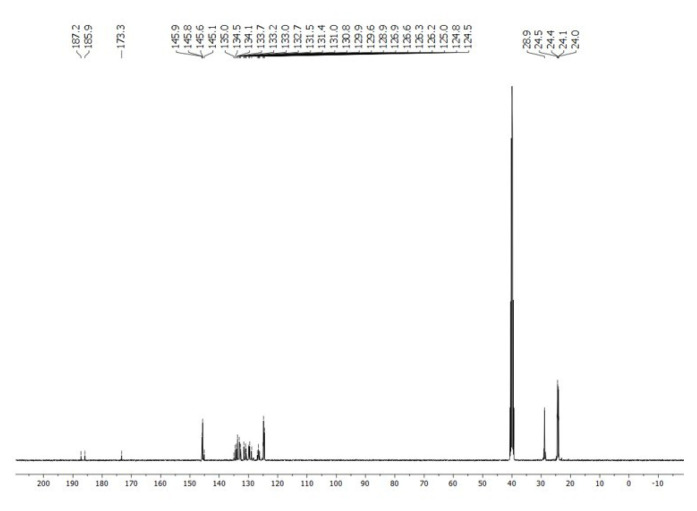
^13^C NMR spectrum of **5** in DMSO-*d*_6_. Multiplet signal at ~40 ppm belongs to DMSO-*d*_6_.

**Figure 13:**
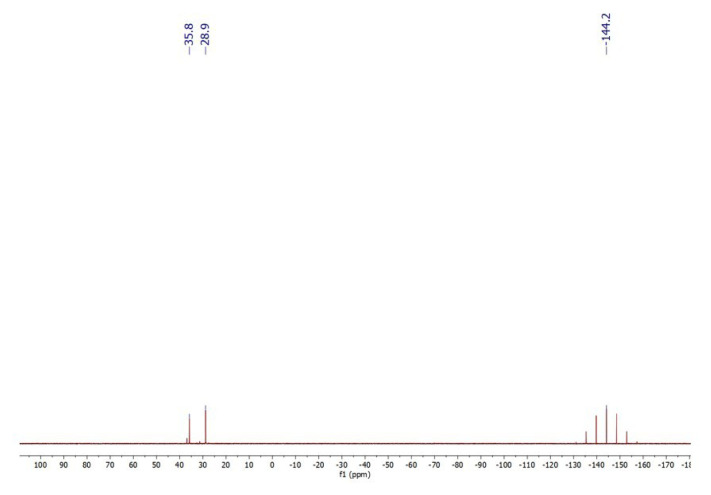
^31^P NMR spectrum of **5** (synthesized in acetone) in DMSO-*d*_6_.

Chloro[1,3-bis(2,6-diisopropylphenyl)imidazol-2-ylidene]gold(I), or (IPr)Au-Cl, could be utilized as a simple synthon for the preparation of mixed NHC and sulfur or phosphine donor ligands, and the resulting heteroleptic Au(I) complexes have shown interesting structural and biological properties [39]. An attempt to prepare an alternative structure with dppm as a bridging ligand gave a binuclear Au(I) complex, **5**, as the major product, which contains only phosphine ligands and no sulfur or NHC donors in the final structure. The ^1^H NMR spectrum of **5** in Figure 10 shows both the starting (IPr)Au-Cl and **5** together with traces of [(IPr_2_)Au]^+^ species. Comparing the derivatization of (IPr)Au-Cl with dppf and dppm ligands reveals that the latter did not result in the expected binuclear (IPr)_2_Au_2_(I)(dppm)_2_ complex with the bridging dppm ligand. Instead, it only formed **5** as the major product, which contains only phosphine donors. Structures similar to **5** with different anions were previously reported, which showed novel photophysical and catalytic properties [40,41]. The molecular structure of **5** is shown in Figure 11. Compound **5** crystallizes by the slow diffusion of diethyl ether into a dichloromethane/methanol solution at room temperature. It crystallizes in the monoclinic space group *P*2/*n*. The Au–P bond lengths and angles are within the reported range of gold(I) thiolate and phosphine complexes [4]. The two Au(I) atoms in **5** (Figure 11) are separated by 2.951(3) Å, which indicates a strong aurophilic interaction. The geometry around each gold atom is close to linear with a P–Au(I)–P angle of 176.07(7)°, and the complex core is close to planarity with a P1–Au1–Au1^i^–P15 torsion angle of 3.07(7)°. This complex was also characterized using ^13^C and ^31^P NMR spectroscopy (Figures 12 and 13).

**Figure 14:**
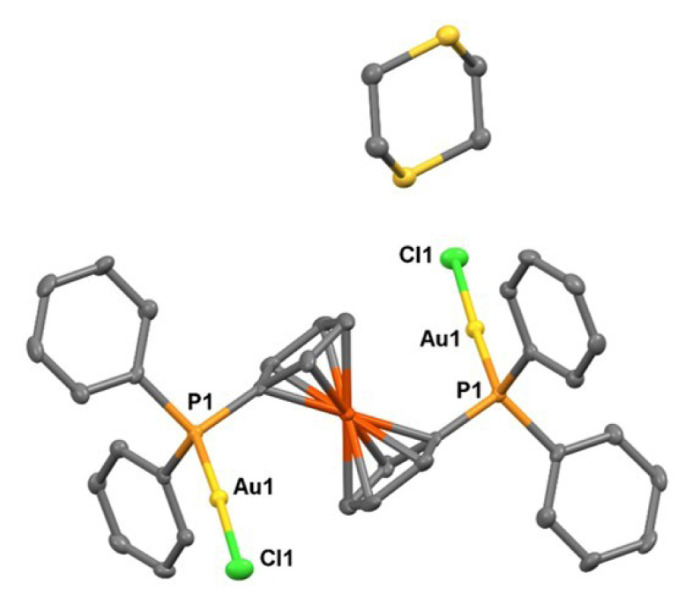
Crystal structure of dppf-dithiane.

Attempts to derivatize (dppf)Au_2_Cl_2_ using both sulfur donor ligands did not produce the intended product. Instead, the starting Au(I) precursor together with uncoordinated 1,3-dithiane or 1,4-dithiane were obtained (Figure 14). Undertaking similar reactions in different solvents such as methanol, acetonitrile, and dichloromethane or a mixture of them did not improve the reaction outcome. Selected structural parameters for **1**, **2**, dppf-dithiane, and **5** are summarized in Supplementary Tables S1 and S2.

While the mechanism by which the dithianes used in this study template the formation of molecular Au(I) assemblies is not fully understood, it is speculated that noncovalent interactions play a crucial role. Understanding these interactions would require further elaborate computational investigations. Noncovalent interactions are fundamental to the design and function of supramolecular and biological systems, encompassing areas such as drug development, catalysis, synthetic chemistry, and crystal engineering [42–45]. Among these relatively weak interactions, those that are of particular significance are hydrogen bonding [46], halogen bonding [47,48], and π–π stacking [49]. These directional forces facilitate the association of discrete entities, including crystallizing molecules, into diverse assemblies, clusters, and supramolecular architectures, thereby enabling the creation of novel functional materials.

## 4. Conclusion

The reactions of dinuclear gold(I)-phosphine precursors with neutral dithiane ligands do not lead to the formation of mixed phosphine/sulfur or NHC/sulfur complexes. Instead, weak sulfur-containing additives act as neutral, noncoordinating structure-directing agents that influence the self-assembly pathways of cationic gold(I)-phosphine units. In the presence of 1,3-dithiane, a trinuclear gold(I) complex featuring a central four-coordinate Au(I) center and terminal T-shaped Au(I) centers is preferentially formed, while removal of the dithiane additive results in an alternative trinuclear isomer that is stable in solution. These closely related assemblies are stabilized by aurophilic interactions, which play a key role in enforcing nonlinear coordination geometries in d^10^ gold(I) systems.

The extension of these reactions to the N-heterocyclic carbene gold(I) precursor further demonstrates the dominance of phosphine ligation and Au···Au interactions, as only phosphine-bridged dinuclear species are obtained. Overall, this study highlights how subtle changes in reaction conditions and the presence or absence of weak, noncoordinating donors can profoundly influence the self-assembly, nuclearity, and structural diversity of multinuclear gold(I) complexes.

## Supporting tables

**Table S1:** Crystal and structure refinement data of 1, 2, and dppf-dithiane.

Identification code	1	2	Dppf-dithiane
Empirical formula	C _50_H _44_Au _3_Cl _2_F _6_P _5_	C _50_H _44_Au _3_Cl _2_F _6_P _5_	C _38_H _36_Au _2_Cl _2_FeP _2_S _2_
Formula weight	1575.6	1575.6	1139.5
*T*, K	120	95	95
*λ*, Å	0.71073	0.71073	1.54184
Crystal system	monoclinic	triclinic	monoclinic
Space group	*P*2 _1_/ *n*	*P -1*	*P*2 _1_/ *n*
*a*, Å	9.4869(2)	15.3788(3)	13.5514(4)
*b*, Å	28.2342(9)	18.7776(3)	8.3789(2)
*c*, Å	18.9606(5)	19.1688(3)	16.4694(4)
*α*, °	90	102.0784(14)	90
*β*, °	95.938(2)	101.1736(15)	103.271(2)
γ, °	90	106.1987(17)	90
*V*, Å ^3^	5051.4(2)	5007.16(17)	1820.09(8)
Z	4	4	2
*ρ* _calc_, g cm ^−3^	2.0717	2.09	2.0791
*μ*, mm ^−1^	9.003	9.1	21.474
*F*(000)	2976	2976	1088
Crystal size, mm	0.298 ′ 0.146 ′ 0.105	0.212 ′ 0.120 ′ 0.066	0.228 ′ 0.030 ′ 0.021
*θ* _min_, *θ* _max_, °	2.28, 29.51	1.93, 29.75	3.82, 73.47
Index ranges	−11<= *h*<=12, −38<= *k*<=35, −26<= *l*<=25	−21<= *h*<=21, −24<= *k*<=26, −26<= *l*<=24	−16<= *h*<=16, −10<= *k*<=8, −20<= *l*<=20
Reflections collected	48684	91651	24974
Independent reflections, *R* _int_	12493, 0.0429	25185, 0.0308	3636, 0.0428
*θ* _full_, ° (completeness 98%)	27.60	27.91	73.47
Data/restraints/parameters	12493/0/595	25185/1043/63	3636/0/215
Goodness-of-fit on *F* ^2^	1.0861	1.2341	1.6986
*R* _1_ [ *I*>3 *σ*( *I*)]	0.0285	0.0305	0.0291
*wR* _2_ [ *I*>3 *σ*( *I*)]	0.0490	0.0796	0.0883
*R* _1_ (all data)	0.0569	0.0427	0.0307
*wR* _2_ (all data)	0.0586	0.0871	0.0893
*Δρ* _min_, *Δρ* _max_, e Å ^−3^	−0.44, 0.50	−0.46, 0.53	−0.67, 1.12

**Table S2:** Crystal and structure refinement data of 5.

Identification code	**5**
Empirical formula	C _52_H _50_Au _2_F _12_ O _2_P _6_
Formula weight	1514.31
*T*, K	95
*λ*, Å	1.54184
Crystal system	monoclinic
Space group	*P*2/ *n*
*a*, Å	10.71460(10)
*b*, Å	9.79770(10)
*c*, Å	24.7792(3)
*α*, °	90
*β*, °	93.4240(10)
γ, °	90
*V*, Å ^3^	2596.64(5)
Z	2
*ρ* _calc_, g cm ^−3^	1.937
*μ*, mm ^−1^	12.958
*F*(000)	1467.671
Crystal size, mm	0.077 ′ 0.062 ′ 0.028
*θ* _min_, *θ* _max_, °	3.574, 73.547
Index ranges	−13<= *h*<=13, 0<= *k*<=12, 0<= *l*<=30
Reflections collected	49363
Independent reflections, *R* _int_	5215, 0.0359
*θ* _full_, ° (completeness 98%)	73.547
Data/restraints/parameters	5215/22/367
Goodness-of-fit on *F* ^2^	1.0200
*R* _1_ [ *I*>3 *σ*( *I*)] *	0.0390
*wR* _2_ [ *I*>3 *σ*( *I*)] *	0.1107
*R* _1_ (all data)	0.0395
*wR* _2_ (all data)	0.1111
*Δρ* _min_, *Δρ* _max_, e Å ^−3^	−1.37, 2.37

* In case of crystal structure 5 I>2σ(I)
